# Surgical Management and Outcomes in Advanced Thyroid Cancer: Insights from a Single-Institution Experience

**DOI:** 10.3390/jcm15124758

**Published:** 2026-06-18

**Authors:** Mario Pacilli, Giovanna Pavone, Elizabeth Khoury, Antonio Ambrosi, Nicola Tartaglia

**Affiliations:** Department of Medical and Surgical Sciences, University of Foggia, 71122 Foggia, Italy; giovanna.pavone@unifg.it (G.P.); elizabeth_khoury.546660@unifg.it (E.K.); antonio.ambrosi@unifg.it (A.A.); nicola.tartaglia@unifg.it (N.T.)

**Keywords:** advanced thyroid cancer, thyroid surgery, anaplastic thyroid carcinoma, surgical decision-making, functional outcomes, quality of life

## Abstract

**Background:** The role of surgery in advanced thyroid cancer remains controversial, particularly in the setting of aggressive tumor behavior, local invasion, and limited therapeutic windows. Advanced thyroid cancer represents a heterogeneous clinical entity that includes anaplastic thyroid carcinoma as well as differentiated and poorly differentiated carcinomas with aggressive features. **Methods:** We conducted a retrospective case series of 10 consecutive patients who underwent surgical management for advanced thyroid cancer at a tertiary referral center over a 30-month period. Clinical presentation, surgical strategy, postoperative complications, adjuvant therapies, and outcomes were analyzed. **Results:** The cohort included 2 papillary, 5 poorly differentiated, and 3 anaplastic thyroid carcinomas. Most patients presented with locally invasive disease and compressive symptoms, including dysphonia and dyspnea. Complete resection (R0) was achieved in five patients and was associated with favorable outcomes, while patients with anaplastic histology experienced poor survival despite palliative interventions. Surgery provided meaningful symptom control in selected patients, particularly those with airway compromise. No perioperative mortality occurred. **Conclusions:** Surgical management of advanced thyroid cancer should be highly individualized and guided by tumor extent, symptom burden, and patient performance status. While surgery alone is insufficient as a standalone treatment, it plays a pivotal role when integrated within a multimodal strategy, offering both oncologic and palliative benefits. Early identification of candidates for surgical intervention and integration with systemic therapies represent key elements in the management of these complex malignancies.

## 1. Introduction

In modern thyroid surgery, increasing emphasis is placed not only on oncological outcomes but also on functional preservation and quality of life. This is particularly relevant in advanced thyroid cancer, where aggressive surgical strategies must be balanced against the risk of complications and functional impairment.

The incidence of thyroid cancer has risen significantly in recent decades, primarily due to advances in diagnostic imaging and the increased detection of small thyroid nodules and microcarcinomas [1].

While most differentiated thyroid carcinomas (DTCs) carry an excellent prognosis, locally invasive disease occurs in 5–15% of cases and is associated with poorer outcomes [2].

Despite being clinically recognizable, there is no universally accepted definition of advanced thyroid cancer. Tumor size, local invasion, and regional spread remain the main determinants, yet preoperative evaluation can be challenging: fine-needle aspiration (FNA) is often inconclusive, making cross-sectional imaging crucial for assessing tumor extension, with molecular markers such as *BRAF*^V600E providing additional diagnostic and prognostic information [3].

Another unresolved issue is the definition of surgically “unresectable” disease. Beyond the technical feasibility of achieving gross tumor resection, operability must also consider the potential morbidity of extensive surgery, particularly when upper aerodigestive tract function is at risk, as well as the patient’s willingness to accept such procedures [4].

Given these uncertainties, the role of surgery in advanced thyroid cancer remains controversial. While aggressive resection may achieve local control and symptom relief, it is often associated with significant morbidity, and the balance between oncologic benefit and quality of life must be carefully considered [5]. High-quality evidence in this area is scarce, with most reports limited to small institutional series.

In this context, we present our single-institution experience with 10 consecutive patients undergoing surgery for advanced thyroid cancer over a 30-month period, focusing on surgical strategies, postoperative outcomes, and the implications for clinical decision-making.

## 2. Materials and Methods

This is a retrospective case series conducted at the General Surgery Unit of the University Hospital of Foggia, a tertiary referral center for endocrine surgery. The study period spanned 30 months, from January 2023 to June 2025.

Patients were eligible for inclusion if they presented with a cervical mass highly suspicious for advanced thyroid cancer and underwent surgical treatment during the study period. Inclusion criteria were based primarily on clinical and radiological features suggestive of advanced thyroid malignancy, including gross extrathyroidal extension involving adjacent structures (e.g., airway, esophagus, vascular structures, or mediastinum), rapidly enlarging or symptomatic cervical masses, and metastatic disease requiring surgical intervention for symptom control.

Preoperative cytological diagnosis was performed in all cases, and all patients underwent preoperative cross-sectional imaging (contrast-enhanced CT and/or MRI) to assess disease extent and resectability. Bronchoscopy and esophagogastroscopy were performed in case of suspected infiltration of the airways or esophagus. Fine-needle aspiration (FNA) often produced inadequate or indeterminate results due to sampling limitations or the undifferentiated nature of the lesion, and thus did not significantly contribute to therapeutic decisions. Molecular profiling was not systematically available for all patients included in this retrospective series and was therefore not analyzed as a study variable. No patients with medullary thyroid carcinoma met the inclusion criteria during the study period. All patients included in the study underwent definitive histopathological diagnosis after surgery.

**Figure 1:**
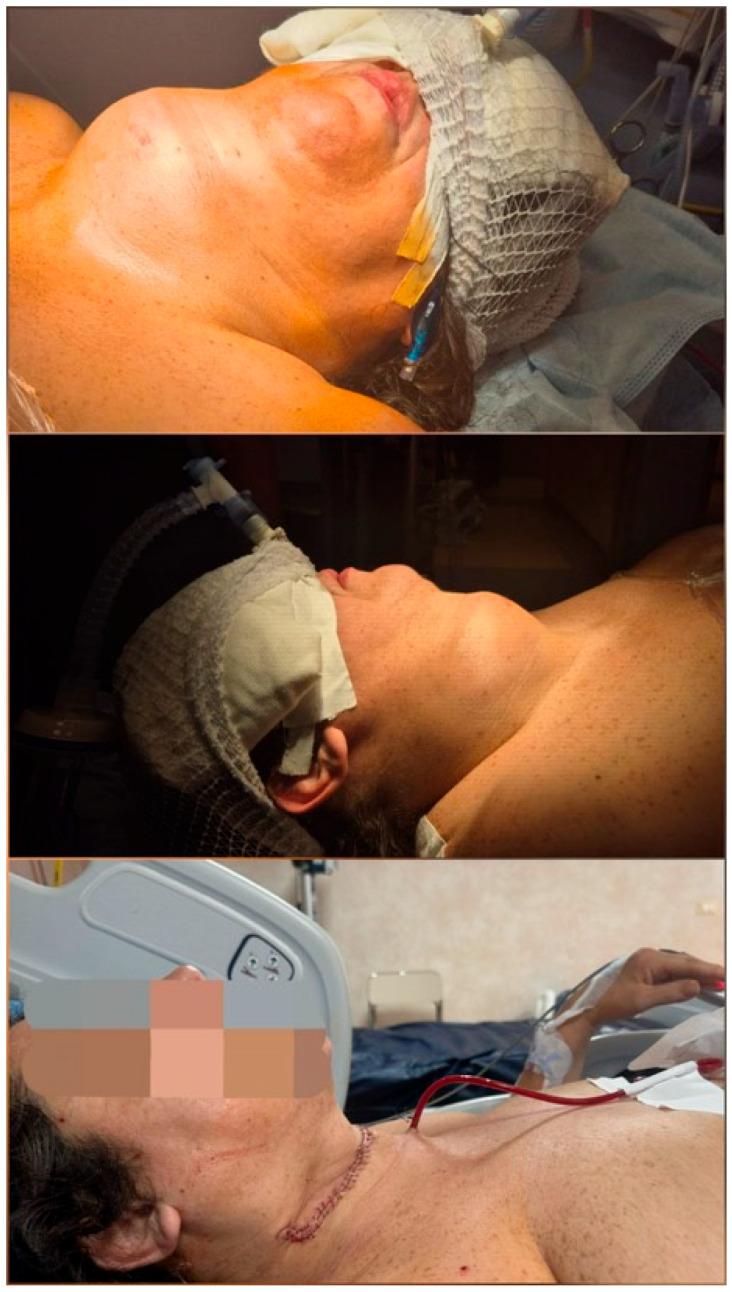
Preoperative (**upper panel**) and postoperative (**lower panel**) clinical appearance of a patient with a massive locally advanced thyroid carcinoma. Orotracheal intubation was not possible because of significant airway distortion, and nasal ventilation was employed. The postoperative image demonstrates a surgical drain placed in the thyroid bed due to the extent of the resection, together with a substantial reduction in cervical enlargement following tumor excision.

Treatment decisions were discussed within a multidisciplinary team including endocrine surgeons, endocrinologists, radiologists, pathologists, radiation oncologists, and medical oncologists. Surgical candidacy was assessed based on disease extent, involvement of adjacent structures, resectability, symptom burden, performance status, and the anticipated impact of surgery on quality of life. The goal of surgery was classified as curative when complete resection appeared feasible and as palliative when symptom control or airway management represented the primary objective (Figure 1).

The primary endpoint was the assessment of surgical feasibility and safety, as determined by intraoperative findings, postoperative complications, and perioperative mortality. Secondary endpoints included oncological and functional outcomes, namely resection margin status (R0, R1, or R2), relief of disease-related symptoms such as airway obstruction, dysphagia, and pain, recurrence during follow-up, and overall survival at the last available assessment.

This study was conducted in accordance with the ethical principles of the Declaration of Helsinki. According to the institutional and national regulations, formal approval by the ethics committee was not required for retrospective case series with anonymized data. All patients provided written informed consent for treatment, and additional consent was obtained for the use of clinical images when applicable.

## 3. Results

During the study period, only ten patients (5 females and 5 males; median age 67 years, range 56–77) met the predefined inclusion criteria for advanced thyroid cancer and underwent surgical management at our institution. No eligible patients were excluded from the analysis (Table 1).

Histological subtypes included papillary thyroid carcinoma (PTC) in 2 patients, poorly differentiated thyroid carcinoma (PDTC) in 5 patients, and anaplastic thyroid carcinoma (ATC) in 3 patients. At presentation, 9 patients had locally invasive disease (T4a or T4b), and 3 patients had distant metastases. Metastatic disease was limited to the lungs in two patients, while one patient presented with both pulmonary and cutaneous metastases. The majority of patients were symptomatic at diagnosis. The most frequent complaints were dysphonia and dyspnea, often associated with stridor or “loud breathing”, reflecting laryngo-tracheal involvement. Other symptoms included dysphagia in 2 patients, persistent cough in 1 patient, and painful cervical swelling in another. Only 2 patients were asymptomatic at presentation, with advanced disease detected through imaging and clinical examination.

The extent of surgery varied according to tumor invasion. Six patients underwent total thyroidectomy with en bloc resection of adjacent structures: two required tracheal resection, one partial esophagectomy, and one vascular resection (resection and reconstruction of the jugular vein). Four patients underwent total thyroidectomy with bilateral neck dissection. In three cases of advanced thyroid cancer, only palliative procedures (biopsy and tracheostomy, or debulking) were possible due to extensive local and systemic disease. R0 resection was achieved in 5 patients, R1 in 2, and R2 in 1 patient, while in 2 patients with ATC, only diagnostic biopsy was performed.

Postoperative complications occurred in 7 patients. The most frequent was transient hypocalcemia (5 cases), followed by permanent recurrent laryngeal nerve palsy (1 case), dysphonia (1 case), and combined hypocalcemia with RLN palsy (1 case).

Neoadjuvant tyrosine kinase inhibitor therapy was administered in two patients with poorly differentiated thyroid carcinoma following multidisciplinary discussion. The absence of significant local symptoms allowed the completion of systemic treatment without delaying urgent surgical intervention. Neoadjuvant therapy achieved meaningful tumor downstaging, improving resectability and reducing the extent of surgery required, thereby potentially avoiding more aggressive procedures involving adjacent structures.

Adjuvant therapies were administered according to tumor type and resection status: radioactive iodine (RAI) was used in 4 patients, external beam radiotherapy (EBRT) in 4 patients, and combined RAI with EBRT in 2 patients. Systemic therapy with tyrosine kinase inhibitors (TKIs) was initiated in 3 patients, all with PDTC or ATC.

The median follow-up was 12 months (range, 1–18 months). At the last follow-up, six patients were alive and disease-free, one patient was alive with persistent disease, and three patients had died of disease, all of whom had anaplastic thyroid carcinoma (ATC). Patients with differentiated and poorly differentiated thyroid carcinomas generally demonstrated favorable outcomes, particularly when complete surgical resection was achieved. All patients who underwent R0 resection remained alive and disease-free at last follow-up, whereas outcomes among patients undergoing R1, R2, or biopsy-only procedures were more heterogeneous and generally reflected the underlying aggressiveness of the disease.

## 4. Discussion

Advanced thyroid cancer represents a clinically heterogeneous group of diseases characterized by aggressive behavior, rapid progression, and frequent invasion of adjacent structures. This category includes not only ATC, but also differentiated (DTC) and poorly differentiated thyroid carcinomas (PDTCs) that exhibit locally advanced growth, compressive symptoms, or resistance to conventional therapies [6]. In this context, the role of surgery is highly debated and must be carefully considered within a multidisciplinary framework. The present case series underscores the complexity of surgical decision-making across this spectrum of advanced thyroid malignancies, rather than focusing on a single histological subtype.

Among advanced thyroid cancers, ATC represents the most aggressive entity, with rapid tumor growth, early metastatic spread, and extremely poor prognosis. The 2021 American Thyroid Association (ATA) guidelines emphasize that surgery for ATC should be undertaken only when complete resection (R0/R1) is technically feasible and not associated with unacceptable morbidity. In most cases, surgery is limited to palliative procedures, particularly for airway protection. The guidelines recommend rapid molecular profiling, as the identification of actionable mutations such as *BRAF*^V600E allows for the use of targeted therapies (e.g., dabrafenib plus trametinib), while external beam radiotherapy remains an option in cases of residual or unresectable disease. Given the poor prognosis, early integration of palliative care is strongly advised [7].

In contrast, advanced DTC and PDTC may share a similarly aggressive clinical presentation, despite a more favorable underlying histology. Surgical intervention remains the cornerstone of initial treatment in patients with resectable thyroid malignancies. In cases of metastatic differentiated thyroid carcinoma (DTC), total thyroidectomy optionally combined with compartment-oriented lymphadenectomy is generally indicated to enable effective radioiodine (^131^I) therapy and to mitigate the risk of locoregional complications associated with tumor progression [8].

There are two cardinal principles in the management and surgical planning of patients with advanced thyroid cancer: assessment of airway patency and timely surgical intervention. An accurate evaluation of disease extent is crucial, as the tumor can exhibit rapid volumetric growth within days, leading to rapid airway compromise, and in some cases, precluding surgical resection [9]. Airway management must therefore be the foremost priority, and should include direct laryngoscopy, with bronchoscopy recommended when tracheal invasion is suspected [10]. Because advanced thyroid cancer typically affects elderly patients, who often present with dysphagia and weight loss, a comprehensive nutritional assessment is also essential; this should include the evaluation of serum albumin and pre-albumin levels. When esophageal invasion is suspected, preoperative esophagogastroscopy should be performed to assess the extent of involvement [11].

Ensuring and maintaining airway patency in advanced thyroid cancer patients can be technically challenging. Routine tracheostomy is not recommended, as it does not improve quality of life or survival, and should be avoided unless there is a clear obstruction to airway patency [12,13]. Tracheostomy limitations are also often related to anatomical distortion and displacement of the trachea caused by large, locally advanced tumors.

The decision to proceed with surgical resection should be based on meticulous preoperative staging with cross-sectional imaging. In patients with locoregionally advanced disease [stage IVa–IVb], surgery should be considered whenever a complete resection (R0 or R1) appears achievable, as gross tumor removal has been associated with improved local control and survival in selected series. For intrathyroidal ATC (stage IVa), total thyroidectomy with central and lateral neck dissection may be indicated, while in selected stage IVb cases, neoadjuvant radiotherapy and/or systemic therapy may allow downstaging and facilitate subsequent resection [14].

Complete tumor excision, rather than debulking, should be the primary surgical objective whenever curative intent is pursued [15,16]. However, the extent of resection must always be balanced against the morbidity of aggressive procedures, such as laryngectomy or esophagectomy. In rare cases of incidental microscopic ATC identified within a DTC specimen, there is currently no evidence to support additional radical surgery, and close radiological surveillance remains the preferred strategy [17,18].

When curative resection is not feasible, surgery may still play a valuable palliative role across the spectrum of advanced thyroid cancers. Limited resections or debulking procedures can alleviate severe symptoms such as airway compromise, dysphagia, bleeding, and pain. In particular, tracheal obstruction represents an emergency scenario in which surgical intervention may be required to prevent respiratory failure. Although non-curative, these procedures can significantly improve patient comfort, preserve functional status, and facilitate access to systemic or radiotherapeutic treatments.

Core needle biopsy may represent a valuable first-line diagnostic tool in patients with suspected anaplastic thyroid carcinoma, often providing sufficient tissue for histopathological and molecular characterization while avoiding unnecessary surgical procedures. Surgical exploration should be reserved for selected cases in which symptom control, assessment of resectability, or palliative intervention is required.

In the context of modern thyroid surgery, the role of advanced technologies such as intraoperative neuromonitoring, and energy-based devices, has gained increasing attention. Although not specifically addressed in this series, these tools may contribute to reducing complication rates and improving functional outcomes, particularly in complex cases requiring extensive dissection. Future integration of such technologies into the management of advanced thyroid cancer may further enhance the balance between oncologic radicality and quality of life.

In our series, surgical decision-making was primarily driven by symptom burden, anatomical extent of disease, and the likelihood of achieving meaningful local control. Patients presenting with airway compromise or significant compressive symptoms generally required immediate surgical evaluation, whereas asymptomatic patients with extensive nodal disease could be considered for neoadjuvant systemic therapy before surgery. These observations highlight the importance of individualized treatment planning in advanced thyroid cancer and reflect the complexity of balancing oncological goals against functional outcomes.

The main limitations of this study are the small sample size, retrospective design, and limited follow-up. Nevertheless, this series provides a real-world representation of surgical decision-making in advanced thyroid cancer, encompassing both anaplastic and aggressive differentiated histologies. A strength of this study is the presentation of real-world surgical management in a heterogeneous cohort of patients with advanced thyroid cancer, including cases requiring complex airway, esophageal, and vascular considerations. The single-institution, single-team experience further ensures consistency in surgical approach and follow-up in this challenging and heterogeneous disease group.

## 5. Conclusions

Advanced thyroid cancer requires a highly individualized multidisciplinary approach, balancing oncological radicality against functional preservation and quality of life. Surgery remains a key component of treatment in selected patients, particularly when complete resection can be achieved. In patients with unresectable disease, palliative surgical procedures may still provide meaningful symptom control and improve quality of life. Given the increasing availability of targeted therapies and multimodal treatment strategies, careful patient selection is essential to optimize outcomes. Further studies with larger cohorts are needed to better define the role of surgery in this challenging clinical setting.

## Figures and Tables

**Table 1:** Patients undergoing surgery for advanced thyroid cancer (PTC, papillary thyroid carcinoma; PDTC, poorly differentiated thyroid carcinoma; ATC, anaplastic thyroid carcinoma; RLN, recurrent laryngeal nerve; TKI, tyrosine kinase inhibitor; RAI, radioactive iodine; EBRT, external beam radiotherapy; AJCC, American Joint Committee on Cancer; R0, microscopically margin-negative resection; R1, microscopically margin-positive resection; R2, macroscopically incomplete resection; N/A, not applicable).

Case	Age/Sex	Histology	Stage [AJCC 8th]	Site of Invasion and/or Metastatic Disease	Symptoms	Surgical Procedure	Resection Margin [R0/R1/R2]	Post-Op Complications	Neo Adjuvant Therapy	Adjuvant Therapy	Follow-Up [Months]	Outcome
**1**	65/M	PTC	T4AN1aM0	Trachea, RLN	Dysphonia, Dyspnea	Total Thyroidectomy + Tracheal Resection	R0	Transient Hypocalcemia	None	RAI + EBRT	18	Alive, Disease-Free
**2**	72/F	PDTC	T4BN0M1	Carotid Sheath, Esophagus	Dysphagia, Dyspnea	Total Thyroidectomy + Partial Esophagectomy	R1	Permanent RLN Palsy	None	EBRT	18	Alive, With Disease
**3**	77/M	ATC	T4BN1bM1	Trachea, RLN, Pulmonary Metastases	Dysphonia, Dyspnea, Stridor, Persistent Cough	Biopsy, Tracheostomy	None	Dysphonia	None	None	None	Death
**4**	58/F	PDTC	T4AN1bM0	Laterocervical Nodes	No Symptoms	Total Thyroidectomy and Bilateral Neck Dissection	R0	Transient Hypocalcemia	TKI	RAI	18	Alive, Disease-Free
**5**	75/M	ATC	T4BN1bM1	Trachea, Esophagus Pulmonary Metastases	Dysphonia, Dyspnea, Stridor	Biopsy, Tracheostomy	None		None	TKI	1	Death
**6**	74/F	ATC	T4BN1bM0	Trachea, Esophagus, Laterocervical Nodes	Dysphagia, Dyspnea	Debulking, Tracheostomy	R2	Hypocalcemia, RLN Palsy		TKI	2	Death
**7**	66/F	PDTC	T4AN1bM0	Laterocervical Nodes	No Symptoms	Total Thyroidectomy and Bilateral Neck Dissection	R0	Transient Hypocalcemia	TKI	RAI	18	Alive, Disease-Free
**8**	59/M	PTC	T4AN1aM0	Trachea, RLN	Dysphonia	Total Thyroidectomy + Tracheal Resection	R0	Transient Hypocalcemia	None	RAI + EBRT	18	Alive, Disease-Free
**9**	66/F	PDTC	T4AN1bM0	Laterocervical Nodes	Painful Lump in The Neck	Total Thyroidectomy and Bilateral Neck Dissection	R0	Transient Hypocalcemia	None	RAI	12	Alive, Disease-Free
**10**	56/F	PDTC	T4BN1bM0	Right Carotid Sheath	Dysphonia, Dyspnea, Stridor	Total Thyroidectomy and Bilateral Neck Dissection + Vascular Resection	R1	Dysphonia and Transient Hypocalcemia		EBRT	6	Alive, Disease Free

## Data Availability

The original contributions presented in this study are included in the article. Further inquiries can be directed to the corresponding author.

## Abbreviations

The following abbreviations are used in this manuscript: ATC: anaplastic thyroid carcinoma. DTC: differentiated thyroid carcinoma. PDTC: poorly differentiated thyroid carcinoma. FNA: fine-needle aspiration. RAI: radioactive iodine. EBRT: external beam radiotherapy. TKI: tyrosine kinase inhibitor. RLN: recurrent laryngeal nerve. PTC, papillary thyroid carcinoma; RLN, recurrent laryngeal nerve.
